# High Yielding, One-Pot Synthesis of Bis(1*H*-indazol-1-yl)methane Catalyzed by 3*d*-Metal Salts

**DOI:** 10.3390/reactions3010005

**Published:** 2022-01-04

**Authors:** Natalie M. Lind, Natalie S. Joe, Brian S. Newell, Aimee M. Morris

**Affiliations:** 1 Department of Chemistry and Biochemistry, Fort Lewis College, 1000 Rim Dr., Durango, CO 81301, USA; 2 Materials and Molecular Analysis Center, Analytical Resource Core, Colorado State University, 200 W. Lake St., Fort Collins, CO 80523, USA

**Keywords:** one-pot, catalysis, bidentate ligand

## Abstract

Synthetic access to poly(indazolyl)methanes has limited their study despite their structural similarity to the highly investigated chelating poly(pyrazolyl)methanes and their potentially important indazole moiety. Herein is presented a high yielding, one-pot synthesis for the 3*d*-metal catalyzed formation of bis(1*H*-indazol-1-yl)methane from 1*H*-indazole utilizing dimethylsulfoxide as the methylene source. Complete characterization of bis(1*H*-indazol-1-yl)methane is given with ^1^H and ^13^C NMR, UV/Vis, FTIR, high resolution mass spectrometry and for the first time, single crystal X-ray diffraction. This simple, inexpensive pathway to yield exclusively bis(1*H*-indazol-1-yl)methane provides synthetic access to further investigate the coordination and potential applications of the family of bis(indazolyl)methanes.

## 1. Introduction

**Figure 1.**
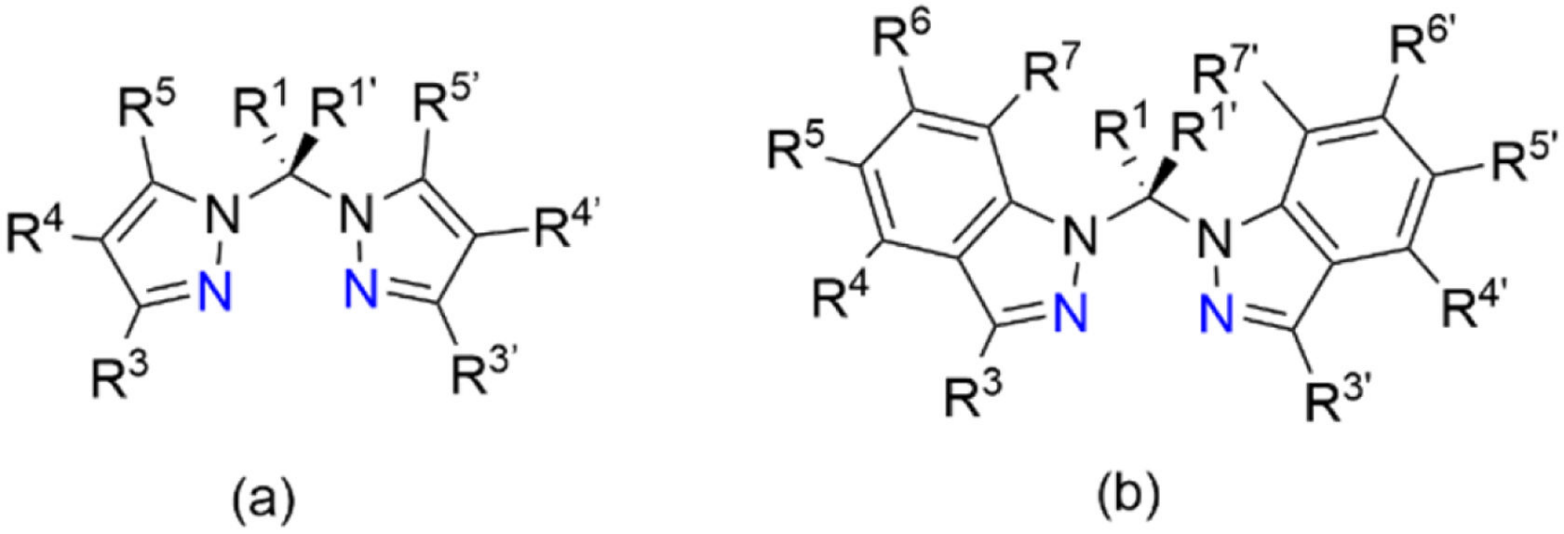
Substitution positions and structural similarities for neutral (**a**) bis(1*H*-pyrazol-1-yl)methane and (**b**) bis(1*H*-indazol-1-yl)methane chelating ligands shown arranged for bidentate binding through the two nitrogen atoms colored in blue. Upon bidentate binding to a metal center, these ligands form six-membered rings with boat conformations.

Poly(1*H*-pyrazol-1-yl)alkanes are a class of ligands that have been investigated due to their overall neutral charge and unique bidentate chelating capabilities through the formation of a six- or seven-membered ring in a boat conformation upon binding to a metal center [1]. The first synthesis of a poly(1*H*-pyrazol-1-yl)methane was reported by Hückel and Chneider in 1937 [2]. Using the potassium salt of pyrazole and chloroform as the carbon source, tri(1*H*-pyrazol-1-yl)methane was prepared [2,3]. In 1970, Trofimenko reported on the synthesis and coordination chemistry of a family of methane and/or pyrazole substituted bis(1*H*-pyrazol-1-yl)methane chelating agents, Figure 1a [3,4]. Synthetically, Trofimenko prepared the substituted pyrazole ligands with methylene bridges by autoclaving the pyrazole and pyrazole salt in the presence of methylene bromide or methylene iodide [4]. A second synthetic method using acetals of aldehydes and ketones was utilized for the preparation of a series of bis(1*H*-pyrazol-1-yl)methane ligands with substituted carbon linkers [4]. The preparation of poly(1*H*-pyrazol-1-yl)methanes utilizing multiple synthetic methods has been extensively studied and recently reviewed [3]. Recent applications of bis(1*H*-pyrazol-1-yl)methanes have highlighted its use in stabilizing chiral coordination complexes [5] and catalysis [6]. Structurally related to the bis(1*H*-pyrazol-1-yl)methane ligands, but less studied are the family of bis(1*H*-indazol-1-yl)methane ligands, Figure 1.

**Scheme 1.**
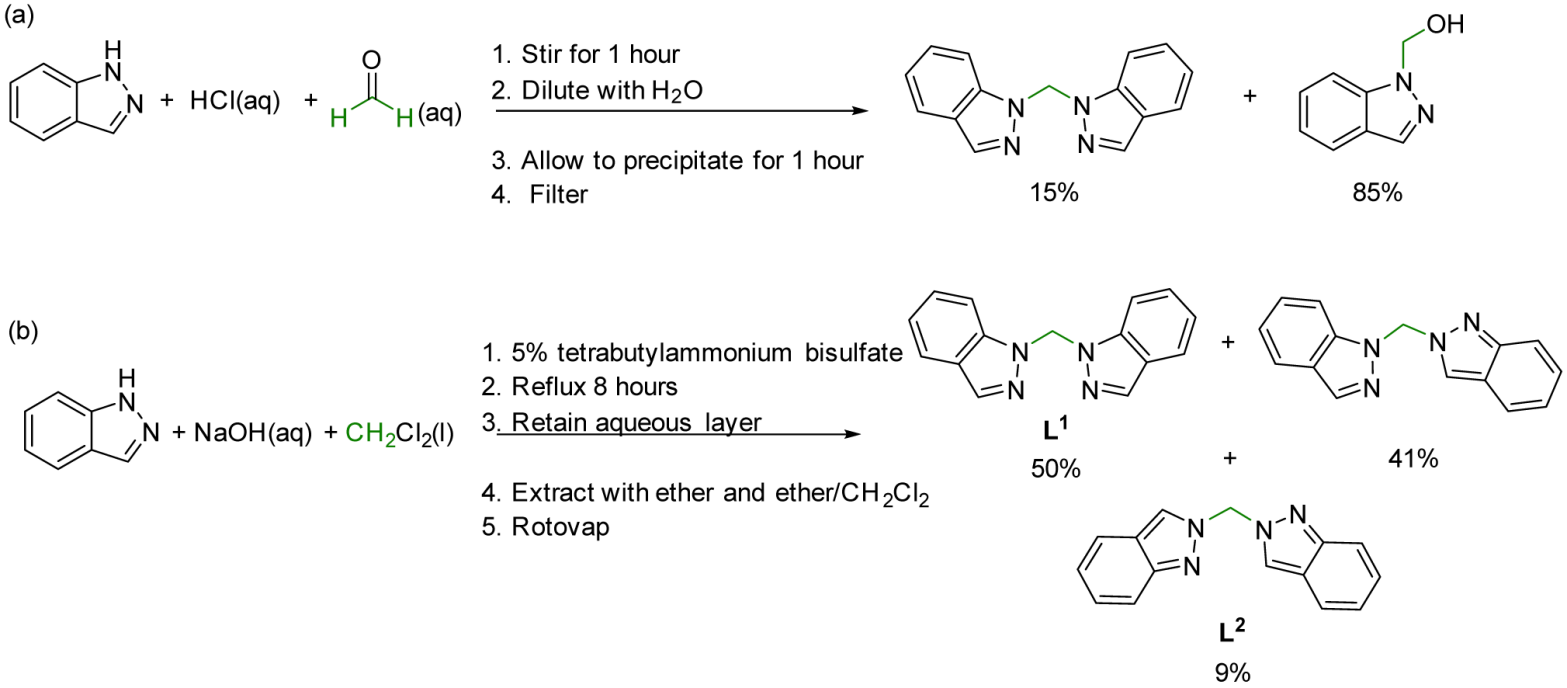
Previously reported one-step synthetic pathways for the formation of bis(1*H*-indazole)methane from 1*H*-indazole with relative amounts of multiple products obtained [7,8]. (**a**) The first synthesis reported in 1964 used formalin, hydrochloric acid and 1*H*-indazole [7] and (**b**) a subsequent synthesis reported in 1982 used methylene chloride, sodium hydroxide and 1*H*-indazole [8]. Both previous syntheses result in a mixture of products.

The synthesis of bis(1*H*-indazol-1-yl)methane was first reported in 1964 as a product mixture with 1-hydroxymethylindazole by combination of 1*H*-indazole with formalin and hydrochloric acid, Scheme 1a [7]. A mixture of bis(1*H*-indazol-1-yl)methane (**L^1^**), (1*H*-indazol-1-yl)(2*H*-indazol-2-yl)methane, and bis(2*H*-indazol-2-yl)methane (**L^2^**) was subsequently reported in 1982 utilizing methylene chloride and phase transfer conditions, Scheme 1b [8]. In 1999, two multi-step synthetic pathways that also required column separation to obtain **L^1^** were reported through the formation of a 2-[(2-acetyoxyethoxy)methyl]indazole intermediate [9].

There was a lapse in the literature for the utilization of bis(1*H*-indazol-1-yl)methane as a chelating ligand despite the significant indazole moiety that has been highly utilized in the pharmaceutical industry [10-12]. In 2010, Pettinari and coworkers utilized the 1982 synthetic method [8] (Scheme 1b) with the first reported separation of **L^1^** and **L^2^** [13]. While the spectroscopic evidence for the separation of **L^1^** and **L^2^** is compelling [13], an extensive search of the literature did not yield any experimental details or separation procedures. Reports with the **L^1^** and **L^2^** chelating ligands including structural and electronic characteristics of more than 50 new coordination complexes has been published since 2010 with binding to group 9, 10, 11, and 12 metal centers [13-15]. However, to our knowledge a high yielding, single step synthesis yielding exclusively the **L^1^** ligand has not yet been reported. Herein, we report optimized reaction conditions for a metal catalyzed one-pot synthesis of bis(1*H*-indazol-1-yl)methane, **L^1^**, using dimethylsulfoxide as the methylene source. Characterization of **L^1^** is detailed using ^1^H and ^13^C NMR, UV/Vis, FTIR, high resolution mass spectrometry, and single crystal X-ray diffraction.

## 2. Materials and Methods

All chemicals were used as received without further purification unless otherwise noted. Cobalt(II) chloride hexahydrate (98–102%) and iron(II) chloride (99.5%, anhydrous) were manufactured by Alfa Aesar (Ward Hill, MA, USA). 1*H*-indazole (98%), iron(II) chloride tetrahydrate (98%), zinc chloride (anhydrous, 99.999%), and nickel(II) chloride hexahydrate (99.9%) were obtained from Sigma-Aldrich (Milwaukee, WI, USA). Dimethyl sulfoxide (DMSO), diethyl ether, methylene chloride, methanol, and acetonitrile were ACS grade and purchased from Fisher Scientific (Waltham, MA, USA).

The ^1^H and ^13^C NMR spectra were collected using a Bruker Ascend spectrometer (Fällanden, Switzerland) operating at 400 and 100 MHz, respectively. DMSO-*d*_6_ (Acros; 99.9% D) was dried over activated molecular sieves and used for all NMR spectra collected. The residual solvent peak was used as the proton and carbon-13 reference. Mass spectra were collected in acetonitrile (anhydrous, 99.8%; Fisher Scientific) on a Thermo-Finnigan LTQ mass spectrometer (San Jose, CA, USA) with ions generated using an electrospray ionization (ESI) source and positive ion mode. UV-Vis solutions were prepared with 1 mM of **L^1^** in methanol and collected on an Agilent Cary 60 UV-Vis spectrophotometer (Santa Clara, CA USA). FTIR of solid-state samples were collected at room temperature from 600–4000 cm^−1^ on a Thermo Scientific Nicolet iS10 with Smart iTR (Madison, WI USA).

### 2.1. *Synthesis and Characterization of Bis(1H-indazol-1-yl)methane*, L^1^

#### 2.1.1. General Procedure for the Formation of L^1^

In a heavy wall cylindrical pressure vessel (15 mL; 150 psig rating, Ace Glass), 1.0 mmol of 1*H*-indazole, 0.01 mmol of metal catalyst, and 1.0 mL of DMSO were combined with a stir bar and sealed with a Teflon screw cap. The loaded reaction vessel was placed in an oil bath at 175 °C and stirred for 24 h. After cooling to room temperature, the crude product was precipitated with 20 mL of deionized water, extracted twice into 50 mL of ethyl acetate, and washed with 10 mL of brine. The organic solvent was removed under reduced pressure and the sample was dried under high vacuum overnight to produce the desired product. Further purification of the product can be accomplished using recrystallization in 50:50 methylene chloride and methanol resulting in a flaky off-white solid product.

#### 2.1.2. Scale-Up Reaction Conditions

A macroscale synthesis of the ligand **L^1^** was accomplished using 0.1 mol of 1*H*-indazole, 1 mmol of CoCl_2_·6H_2_O, and 10 mL of DMSO in a 50 mL round-bottom flask with a water cooled-condenser. A reflux temperature of 175 °C with increased reaction time was needed for complete conversion of 1*H*-indazole to **L^1^** which can be monitored by TLC using 50:50 ethyl acetate and *n*-hexanes. The precipitation and workup of the product were consistent with the general procedure using a 10X scaling factor.

#### 2.1.3. Characterization of L^1^

Formation of **L^1^** was monitored by ^1^H NMR in DMSO-*d*_6_ for all reactions screened. ^1^H NMR (400 MHz, DMSO-*d*_6_, 295 K) δ: 8.12 (d, 1H, H^3/3′^, *J* = 0.9 Hz), 7.96 (dd, 1H, H^7/7′^, *J* = 8.5 and 0.9 Hz), 7.74 (dt, 1H, H^4/4′^, *J* = 8.1 and 1.0 Hz), 7.46 (ddd, 1H, H^6/6′^, *J* = 7.7, 1.3, and 1.1 Hz), 7.17 (ddd, 1H, H^5/5′^, *J* = 7.5, 1.0, and 0.8 Hz), 7.10 (s, 1H, CH_2_), see Figure S1 of the Supporting Information. Further characterization was undertaken to verify the assigned structure to **L^1^**. ^13^C{^1^H} NMR (100 MHz, DMSO-*d*_6_, 295 K) δ: 139.7 (C_7a_), 134.9 (C_3_), 127.2 (C_6_), 124.5 (C_3a_), 121.7 (C_5_), 121.4 (C_4_), 110.8 (C_7_), and 60.6 (CH_2_); see Figure S2 of the Supporting Information. HRMS (ESI): *m/z* 249.1138 [**L^1^** + H]^+^ and 131.0603 [C_7_H_5_N_2_ + CH_2_]^+^. UV/Vis λ_max_ (log ε): 251 (3.54), 260 (3.51), 288 (3.52), 293 (3.52), 299 (3.50). IR (cm^−1^): 3106 (w), 3060 (m), 2957 (w), 2921 (w), 1617 (m), 1499 (m), 1463 (m), 1439 (w), 1353 (m), 1279 (m), 1197 (s), 1150 (w), 1005 (m), 906 (m), 828 (m), 758 (s), 736 (s).

### 2.2. X-ray Crystallography

#### 2.2.1. Experimental Conditions for Single Crystals Growth

Reaction conditions consisting of 3.1 mmol of 1*H*-indazole and 0.31 mmol of *trans*-dichlorotetrakis(pyridine)cobalt(III) chloride [16] were dissolved in 2.6 mL of DMSO. After refluxing at 175 °C for 24 h, the reaction mixture was poured into a beaker and allowed to concentrate at room temperature. After one week, long needle-like crystals were observed in a dark green mother liquor. The crude product was filtered and washed with DMSO and diethyl ether. After overnight vacuum drying, single crystals suitable for X-ray diffraction were obtained from recrystallization in methylene chloride.

#### 2.2.2. X-ray Crystallography Data Collection

The structure was determined for the compound listed in Table 1. Single crystals were coated with Paratone-N oil and mounted under a cold stream of dinitrogen gas. Single crystal X-ray diffraction data were acquired on a Bruker Kappa APEXII diffractometer (Karlsruhe, Germany) equipped with a CCD detector and a graphite monochromator using Mo Kα radiation (λ = 0.71073 Å). Initial lattice parameters were obtained from a least-squares analysis of more than 100 reflections; these parameters were later refined against all data. The crystals did not show significant decay during data collection. Data were integrated and corrected for Lorentz and polarization effects using Bruker APEX4 software [17], and semiempirical absorption corrections were applied using SCALE [18]. Space group assignments were based on systematic absences, E statistics, and successful refinement of the structures. Structures were solved using Direct Methods and were refined with the aid of successive Fourier difference maps against all data using the SHELXT 6.14 software package [19]. Thermal parameters for all non-hydrogen atoms were refined anisotropically. All hydrogen atoms were assigned to ideal positions and refined using a riding model with an isotropic thermal parameter 1.2 times that of the attached carbon atom (1.5 times for methyl hydrogens). All other metric parameters can be found in deposited crystal structure file.

## 3. Results

### 3.1. *One-Pot Synthesis of Bis(1H-indazol-1-yl)methane*, L^1^

**Scheme 2.**
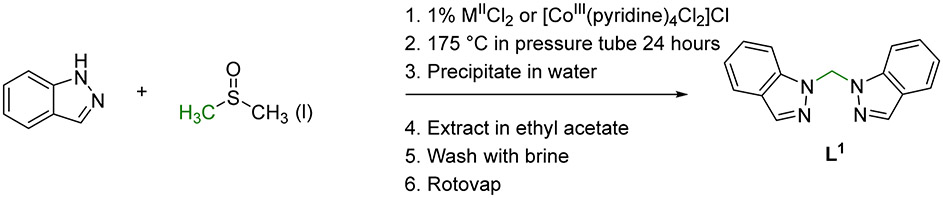
One-pot synthetic pathway and workup for the formation of **L^1^**.

The generalized synthetic pathway for the optimized high yielding, one-pot synthesis forming exclusively **L^1^** from 1*H*-indazole and dimethylsulfoxide using either a commercially available 3*d*-metal salt catalyst or a synthetic Co(III) coordination complex is summarized in Scheme 2. Following the 24 h heating at 175 °C in a pressure tube, the desired product **L^1^** was precipitated from the DMSO reaction solution with deionized water. **L^1^** was then extracted into ethyl acetate and washed with brine. Collection of the organic layer allowed for concentration by evaporation followed by overnight drying on a high vacuum line to yield the desired, pure **L^1^** product which was verified in each reaction by ^1^H NMR.

Milder reaction conditions were tested using several other potential methylene source solvents near their boiling points in the presence of 10% CoCl_2_·6H_2_O including dimethylformamide, toluene, dibromomethane, acetonitrile, ethanol and dichloromethane. However, no product formation was observed from any solvent other than DMSO and only the starting material 1*H*-indazole was detected at the end of the 24 h reaction. Attempts at addition one or two molar equivalents of DMSO in toluene were also unsuccessful in forming **L^1^**. For a full listing of solvents and reaction details of conditions tested, see Table S4 of the Supplemental Information.

Time optimization studies were also undertaken to find the minimum reaction time needed to fully convert 1*H*-indazole to the desired **L^1^** in DMSO. Using a 1% catalyst loading of CoCl_2_·6H_2_O, reactions under our standard conditions were stopped and worked up at 8, 12, 16, 20, 24, and 48 h. The reactions at 16 h and earlier all showed no conversion to the desired **L^1^** and only 1*H*-indazole was detected by ^1^H NMR. At 20 h, a mixture of 1*H*-indazole and **L^1^** were present. Both the 24 and 48 h reactions showed complete conversion to the desired dimer product in similar yields giving the optimized reaction time of 24 h.

Moving forward with the DMSO solvent/methylene source and optimized reaction time, control reactions in the absence of a metal salt showed that dimer formation only partially occurs in the 24 h reaction window but a significant amount of 1*H*-indazole still remained, entry 1 of Table 2. Increasing the control reaction time does result in increased conversion of 1*H*-indazole into **L^1^** along with multiple other products detected by ^1^H NMR that could not be easily separated (entry 2). Further reaction optimization revealed that a 1% catalyst loading of commercially available CoCl_2_, FeCl_2_, NiCl_2_, or ZnCl_2_ all resulted in high yields of the desired product **L^1^** (entries 5, 7, 9–12) in 24 h. Regardless of the metal salt used, all the reactions took a minimum of 24 h for full conversion to **L^1^**. Hydrated as well as anhydrous forms of the cobalt(II) and iron(II) chloride salts both produced very similar results with the anhydrous forms giving slightly lower yields (entries 5 and 7; 9 and 10). *Trans*-[Co(pyridine)_4_Cl_2_]Cl was synthesized from the previous literature [16] and tested as a Co(III) catalyst (entry 8) which showed similar yields and product formation to the commercially available cobalt(II) chloride.

A macroscale reaction for the production of **L^1^** was also tested for future applications. A high yielding gram-scale reaction was found with slight modification using a reflux setup to accommodate the increased amounts of starting materials and an increased reaction time which can be monitored by TLC for the complete conversion of 1*H*-indazole to **L^1^**. The scale-up tested had a consistently high yield of pure **L^1^** product using a 1% CoCl_2_·6H_2_O catalyst providing evidence of scalability for this one-pot synthesis.

### 3.2. *Characterization of Bis(1H-indazol-1-yl)methane*, L^1^

**Figure 2.**
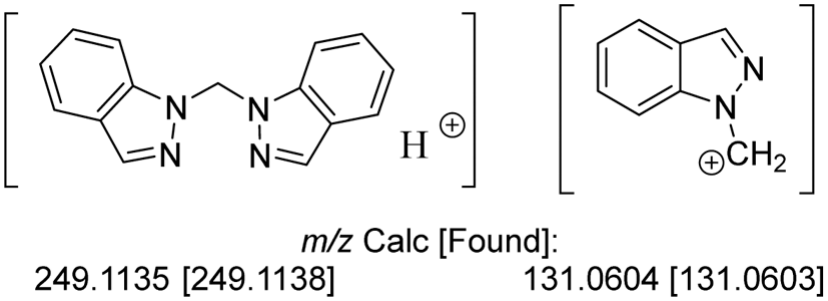
High resolution ESI mass spectrometry parent and fragment ions observed for **L^1^**.

^1^H and ^13^C NMR spectra have been previously reported for **L^1^** [8,9,13] and were consistent with the chemical shifts and splitting patterns allowing for assignment of the methylene bridged dimer structure. A comparison table of the NMR data collected herein and previously reported in the literature is shown in Table S1 of the Supporting Information. UV/Vis data for **L^1^** have also been previously determined [9] and were used to support the assigned structure of **L^1^** as bis(1*H*-indazol-1-yl)methane. Key IR vibrations corresponding to the ν(C=C) and ν(C=N) at 1617 and 1499 cm^-1^ were observed for the **L^1^** reported herein and are consistent with those previously reported [13]. Further support for the structure of **L^1^** was obtained through high resolution ESI mass spectrometry which showed the expected parent ion as well as a monomeric fragment, Figure 2.

**Figure 3.**
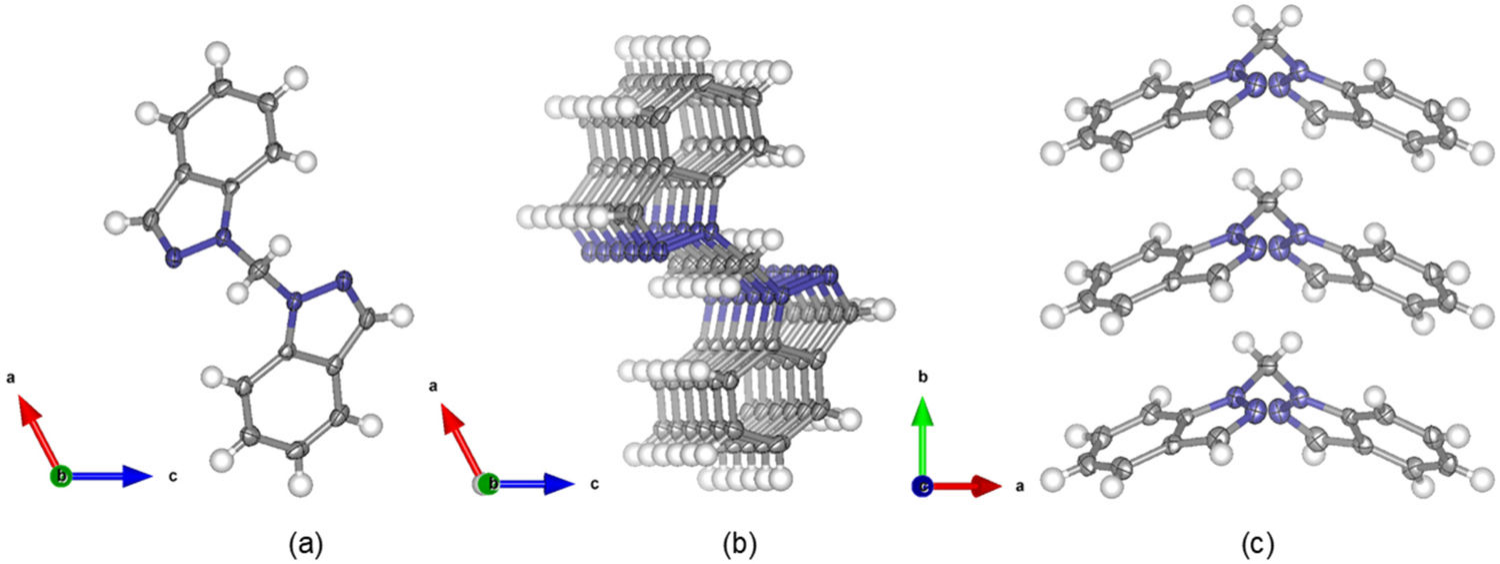
Crystal structure of bis(1*H*-indazol-1-yl)methane, **L^1^** with thermal ellipsoids displayed at 50% probability (**a**) viewed down the *b*-axis. Extended packing viewed (**b**) with a 5° rotation about the crystallographic *b*-axis and (**c**) down the crystallographic *c*-axis.

Single crystal X-ray structures with **L^1^** bound to metal centers have been reported [12-14], but a single crystal structure of **L^1^** could not be found in any crystallographic databases. Our structure determination revealed that **L^1^** crystallizes in the monoclinic space group C_2_. No significant bond distances or bond angles within each indazole moiety of **L^1^** differ significantly from the recently reported structure of 1*H*-indazole [20]. A 50% probability thermal ellipsoid plot of the single crystal structure of **L^1^** is presented in Figure 3a with a N1-CH_2_-N1′ bond angle of 113.5(3)°. Extended pi-stacking in the crystallized structure can be viewed in Figure 3b,c with 4.0856 Å distances between the benzene rings.

## 4. Discussion

### 4.1. *One-Pot Synthesis of Bis(1H-indazol-1-yl)methane*, **L^1^**

The optimized one-pot reaction conditions presented herein provide a high yielding, simple synthetic pathway for the formation of the single product **L^1^** that can be utilized in chelating ligand applications. The dimerization and addition of a methylene group occur during the 24 h high temperature reaction where a commercially available metal(II) chloride salt or a Co(III) coordination complex catalyze the transformation of 1*H*-indazole. The use of DMSO as a methylene source in organic reactions has been recently reported [21-27], and this one-pot synthesis provides another example. Interestingly, no other solvent tested near its boiling point (DMF, toluene, CH_2_Br_2_, CH_3_CN, CH_3_CH_2_OH, CH_2_Cl_2_) in the presence of the CoCl_2_·6H_2_O catalyst was able to act as a methylene source and convert 1*H*-indazole to the desired **L^1^** product. This suggests that the 175 °C temperature and/or high molar excess of DMSO along with a metal catalyst was necessary to remove protons from N1 and N1′, insert a CH_2_ group and dimerize 1*H*-indazole. Using the high temperature conditions over 24 h suggests that a thermodynamic product of exclusively **L^1^** is formed and not the mixture of products that have been previously observed [7,8], Scheme 1. This observation is also consistent with the significantly more stable 1*H*- vs. 2*H*-indazole tautomer [28,29]. This inexpensive one-pot synthesis provides access to the **L^1^** ligand for future development in chelating applications and potentially in the development of new medicinal compounds.

### 4.2. *Crystal Structure of Bis(1H-indazol-1-yl)methane*, L^1^

**Figure 4.**
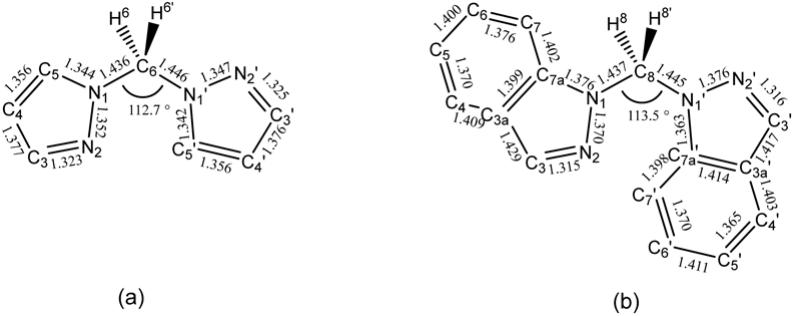
Select bond lengths and angles of (**a**) bis(1*H*-pyrazol-1-yl)methane [30] and (**b**) **L^1^**.

There are significant structural similarities between the known structure of the related bis(1*H*-pyrazol-1-yl)methane [30] and **L^1^** as highlighted with selected bond lengths and angles in Figure 4. Full tables of bond lengths and angles are given in Tables S2 and S3 of the Supporting Information. Some differences between these ligands are of note. While the bond lengths between N1/N1′ and the methylene group are within experimental error between the two ligands, there is a 0.8° increase in bond angle between the N1—CH_2_—N1′ in **L^1^** that is likely the result of increased steric hinderance and differing electronic properties. The indazole moiety causes extended pi-stacking networks in **L^1^** as viewed in Figure 3b,c that are not present in bis(1*H*-pyrazol-1-yl)methane. In comparison to bis(1*H*-pyrazol-1-yl)methane, **L^1^** also shows longer bond lengths throughout its pyrazole moiety except for N2/2′—C3/3′ where the bond lengths are compressed in **L^1^**. This trend within the pyrazole moiety may be explained by Clar’s aromatic π-sextet rule [31] and is consistent with the bond lengths observed in the crystal structures of 1*H*-pyrazole [32] and 1*H*-indazole [20].

Comparing the crystal structure of **L^1^** to published structures of **L^1^** coordinated to group 9, 10, 11 and 12 metal centers [13-15] reveals that significant rotation in solution around the C—N1/1′ bond is necessary to create bidentate bonding to metal centers through N2 and N2′. This could explain why **L^1^** has been observed to act as a bridging ligand in bi- or poly-metallic Ag(I) coordination complexes [14]. It has also been observed that the decreased basicity [33] and steric differences of **L^1^** in comparison to bis(1*H*-pyrazol-1-yl)methane allow for different stoichiometric coordination complexes to be formed [13] opening up new libraries of compounds and potentially new applications. Finally, the addition of the aromatic benzene ring to the pyrazole moiety in **L^1^** shows that coordination complexes with **L^1^** can also have extended pi-stacking networks [15] as was observed with the ligand alone. These steric and electronic differences for **L^1^** could provide insights in future synthetic designs and application studies.

### 4.3. *Mechanistic Studies and Future Applications of* L^1^

The optimized reaction conditions provide some preliminary mechanistic evidence for the formation of **L^1^** from 1*H*-indazole. The use of DMSO as the methylene source in similar reactions has been observed [21-27]. In some cases, the DMSO acts both as the methylene source and an oxidant in the presence of metals [24] and in other cases metal free catalysis is observed with DMSO and a strong oxidant [27]. In order to test if a strong oxidant might be involved in the conversion of 1*H*-indazole to **L^1^** in DMSO, hydrogen peroxide was added in the absence of a metal catalyst (entry 3 of Table 2). However, there was no formation of the desired **L^1^** under these reaction conditions. Even in the presence of a metal catalyst with H_2_O_2_, still no product formation was observed, entry 6 of Table 2. Our results indicate that a metal catalyst with DMSO at 175 °C was necessary to produce high yields of the desired **L^1^**. Time optimization studies also provide insight into the mechanism where a long induction period (16 < *t* < 20 h) was observed with only 1*H*-indazole being detected with the CoCl_2_ catalyst in DMSO at 16 h before production of the desired methylene dimer started to be detected at 20 h. A variety of metal catalysts in hydrated or anhydrous forms, in +2- or +3-oxidation states, and metals with more or less accessible redox states were all able to successfully complete the insertion of the methylene group and dimerization suggesting that the metal catalyst might act as a Lewis acid. Further support is given by the minimum reaction time for the formation of **L^1^** being 24 h regardless of the 3*d* metal salt used. This finding of the metal salt acting as a Lewis acid is consistent with a previously postulated mechanism for the formation of methylenebisamides using DMSO as both a proposed methylene source and an oxidant in the presence of a NiCl_2_ catalyst for dimerization [24]. Further studies are needed to determine the mechanism of formation for **L^1^** as well as related product formations considering the growth of literature utilizing DMSO as a methylene source in the presence of trace metal catalysts [23,24,26]. Further insights into the mechanism would continue to allow for reaction optimization for creating a family of substituted bis(1*H*-indazol-1-yl)methane complexes and exploring their potential applications.

## 5. Conclusions

This article presents on a simple, high yielding one-pot synthesis producing exclusively bis(1*H*-indazol-1-yl)methane (**L^1^**) from 1*H*-indazole and DMSO catalyzed by 1% of any of the following metal salts: CoCl_2_, FeCl_2_, NiCl_2_, ZnCl_2_, or *trans*-[Co(pyridine)_4_Cl_2_]Cl. Complete characterization of **L^1^** is presented including ^1^H and ^13^C NMR, UV/Vis, FTIR, high resolution mass spectrometry, and for the first time single crystal X-ray diffraction. Creating synthetic access and diffraction data will allow for expansion of our knowledge on the bis(1*H*-indazol-1-yl)methane family of ligands to be utilized in future synthetic, mechanistic, chelating and pharmaceutical applications.

## Supplementary Material

Supplementary Information

## Figures and Tables

**Table 1.** Crystal data and details of refinement for **L^1^**.

Parameters	**L^1^**
Formula	C_15_H_12_N_4_
Formula Weight (g/mol)	248.29
Temperature	100(2) K
Crystal system	Monoclinic
space group, Z	C_2_, 4
a (Å)	23.6129(19)
b (Å)	4.0856(4)
c (Å)	13.9334(12)
α (°)	90
β (°)	117.743(5)
γ (°)	90
Volume (Å^3^)	1189.67(19)
ρ (g/cm^3^)	1.3806
*R*_1_^a^	0.0863
*wR*_2_^a^	0.0909
GOF	1.037

a Definitions of *R*_1_ and *wR*_2_. *R*_1_ = Σ ∣ ∣*F*_o_∣ – ∣*F_c_*∣ ∣ /Σ ∣*F_o_*∣; *wR*_2_ = {[*w*(*F_o_*^2^ – *F_c_*^2^)^2^]/[*w*(*F_o_*^2^)^2^]}^1/2^.

**Table 2.** Reaction screening conditions for the formation of **L^1^** from 1*H*-indazole and DMSO as the methylene source. All reactions were run at 175 °C in high pressure reaction test tubes.

Entry	Time (h)	Metal Catalyst	Additive	Dimer Formation?	Isolated Yield ^a^
1	24	-	-	Partial	-
2	72	-	-	Yes ^b^	- ^b^
3	24	-	H_2_O_2_	No	-
4	24	10% CoCl_2_·6H_2_O	-	Yes	94%
5	24	1% CoCl_2_·6H_2_O	-	Yes	96%
6	24	1% CoCl_2_·6H_2_O	H_2_O_2_	No	-
7	24	1% CoCl_2_	-	Yes	92%
8	24	1% [Co(pyridine)_4_Cl_2_]Cl	-	Yes	96%
9	24	1% FeCl_2_·4H_2_O	-	Yes	98%
10	24	1% FeCl_2_	-	Yes	96%
11	24	1% NiCl_2_·6H_2_O	-	Yes	96%
12	24	1% ZnCl_2_	-	Yes	94%

a Average isolated yield of duplicate experiments.

b Evidence of dimer formation is present in the ^1^H NMR. However, there is a mixture of products present and attempts at isolation of the dimer were unsuccessful.

## Data Availability

The data presented in this study are available in the main text and Supplementary Materials.
