# Supplementary material for: High Yielding, One-Pot Synthesis of Bis(1H-indazol-1-yl)methane Catalyzed by 3d-Metal Salts
Source: Reactions (Basel). Author manuscript; Available in PMC 2022 Mar 1. (PMC8779710; doi:10.3390/reactions3010005)
Supplement: Supplementary Information [file NIHMS1768802-supplement-Supplementary_Information.pdf]

Supplementary Information for:

Article

High Yielding, One-Pot Synthesis of Bis(1*H*-Indazol-1-yl)methane Catalyzed by 3*d*-Metal Salts

Natalie M. Lind, Natalie S. Joe, Brian S. Newell and Aimee M. Morris

Table S1. ^{1}H and ^{13}C NMR data collected in this study for **L^1** and compared to the previous literature. All measurements in DMSO-d_6 at 100 and 400 MHz for ^{13}C and ^{1}H respectively unless otherwise noted.

Name	Structure	^{13}C chemical shifts	^{1}H chemical shifts	Ref
1*H*-indazole	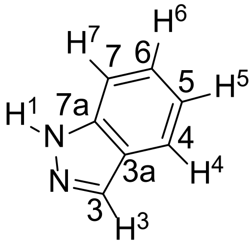	C3: 133.4<br>C3a: 122.8<br>C4: 120.4<br>C5: 120.1<br>C6: 125.8<br>C7: 110.0<br>C7a: 139.9	H^1: 13.01 (s, 1H)<br>H^3: 8.05 (s, 1H)<br>H^4: 7.75 (d, 1H)<br>H^5: 7.09 (t, 1H)<br>H^6: 7.33 (t, 1H)<br>H^7: 7.52 (d, 1H)	[1, 2]
bis(1*H*-indazol-1-yl)methane[a]	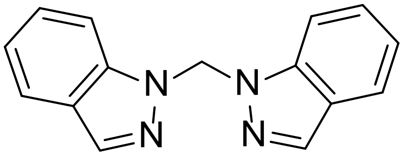	CH_2: 60.1<br>C3: 134.4<br>C3a: 122.3<br>C4: 121.4[b]<br>C5: 121.0[b]<br>C6: 127.0<br>C7: 110.0<br>C7a: 139.6	CH_2: 7.08 (s, 1H)<br>H^3: 8.09 (s, 1H)<br>H^4: 7.71 (d, 1H)<br>H^5: 7.14 (t, 1H)<br>H^6: 7.44 (t, 1H)<br>H^7: 7.93 (d, 1H)	[3, 4]
bis(1*H*-indazol-1-yl)methane[c]	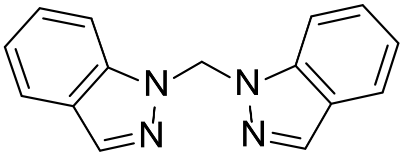	CH_2: 60.6<br>C3: 134.9<br>C3a: 124.5<br>C4: 121.4<br>C5: 121.7<br>C6: 127.2<br>C7: 110.8<br>C7a: 139.7	CH_2: 7.10 (s, 1H)<br>H^3: 8.12 (d, 1H)<br>H^4: 7.74 (dt, 1H)<br>H^5: 7.17 (ddd, 1H)<br>H^6: 7.46 (ddd, 1H)<br>H^7: 7.96 (dd, 1H)	This work

[a] The ^{13}C NMR were measured in $CDCl_3$ at 15.1 MHz; formal assignments were not listed for the proton NMR signals.
[b] Original publication notes that the assignment of these two carbon atoms may be reversed.
[c] The ^{1}H and ^{13}C signal assignments were confirmed through COSY and HSQC.

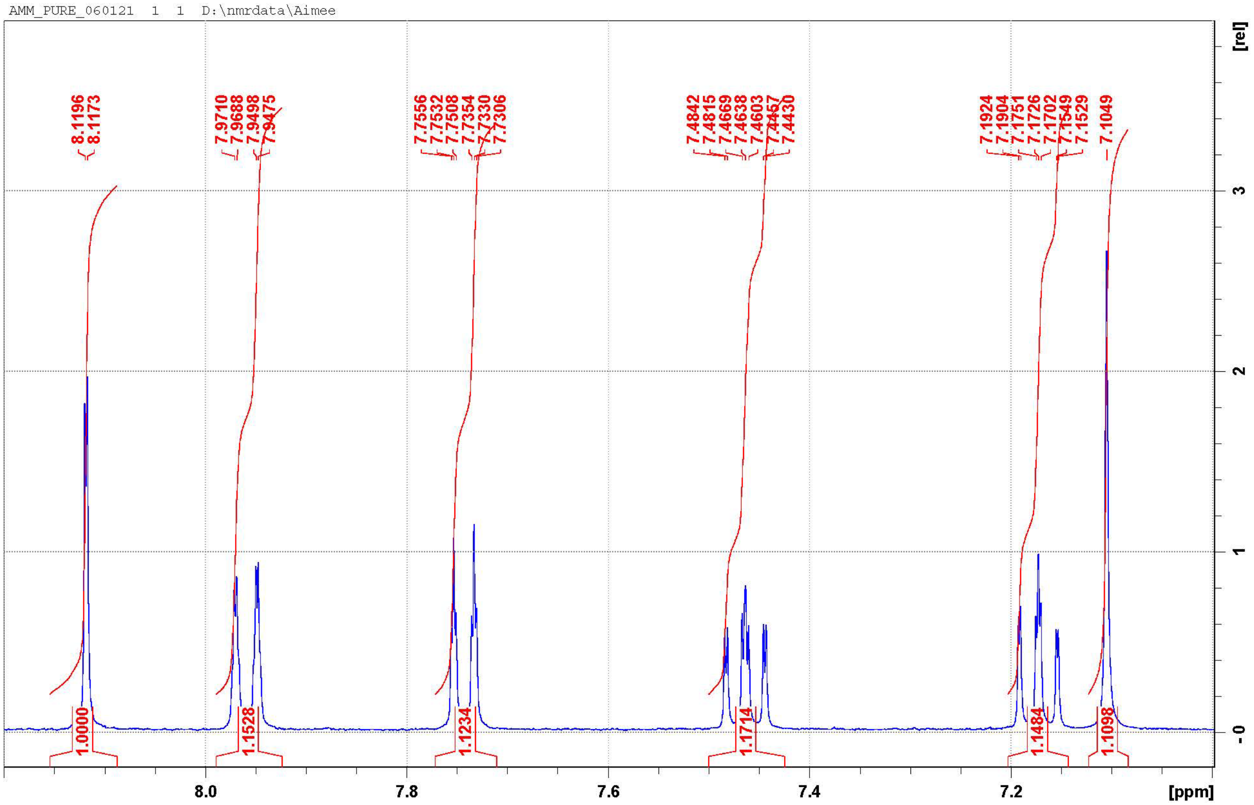

Figure S1. ^{1}H NMR of bis(1*H*-indazol-1-yl)methane (**L**^{1}) in DMSO-d_6.

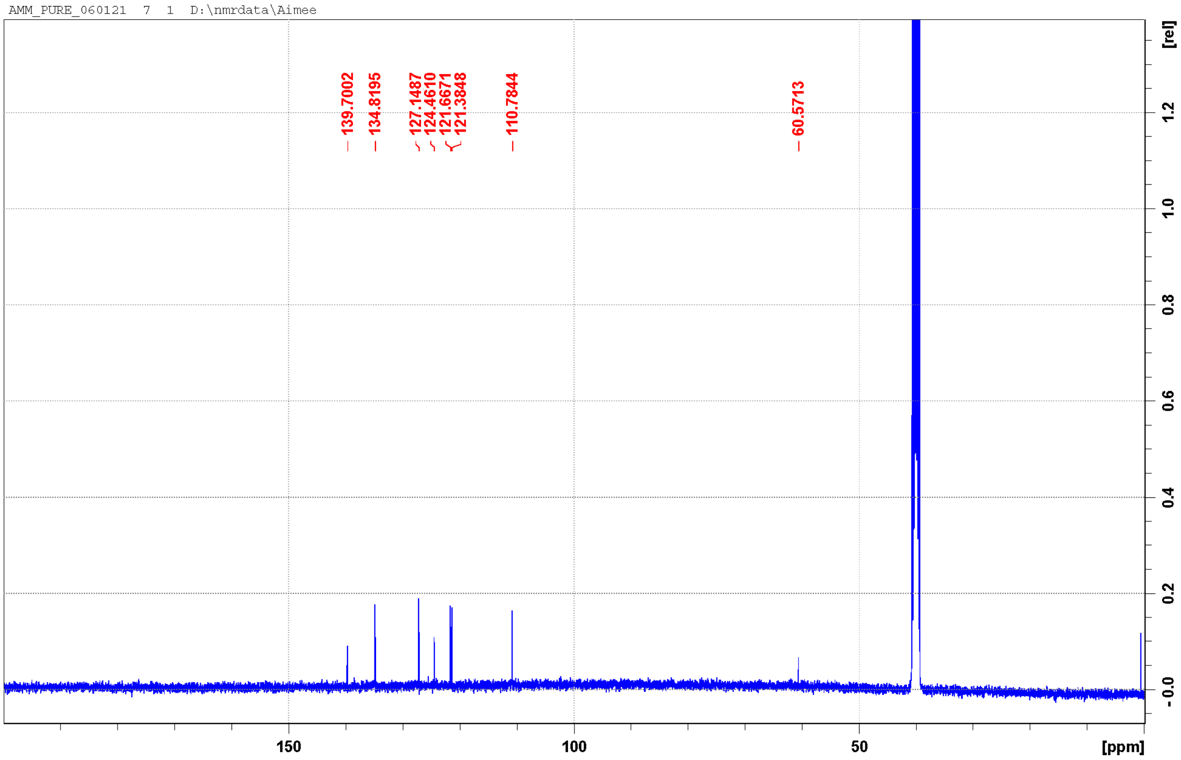

Figure S2. ^{13}C NMR of bis(1*H*-indazol-1-yl)methane (**L**^{1}) in DMSO-d_6.

Table S2. Crystallographic Table of Bond Lengths (Å) for **L^1**

N1_1-N2_1	1.370(3)	N1_1-C7A_1	1.376(3)
N1_1-C8_1	1.437(3)	N2_1-C3_1	1.315(3)
C3_1-C3A_1	1.429(4)	C3_1-H3_1	0.950000
C3A_1-C7A_1	1.399(4)	C3A_1-C4_1	1.409(4)
C4_1-C5_1	1.370(4)	C4_1-H4_1	0.950000
C5_1-C6_1	1.400(4)	C5_1-H5_1	0.950000
C6_1-C7_1	1.376(4)	C6_1-H6_1	0.950000
C7_1-C7A_1	1.402(3)	C7_1-H7_1	0.950000
C8_1-H8A_1	0.990000	C8_1-H8B_1	0.990000
N9_2-C15A_2	1.363(3)	N9_2-N10_2	1.376(3)
N9_2-C16_2	1.445(3)	N10_2-C11_2	1.316(4)
C11_2-C11A_2	1.417(4)	C11_2-H11_2	0.950000
C11A_2-C12_2	1.403(4)	C11A_2-C15A_2	1.414(4)
C12_2-C13_2	1.365(4)	C12_2-H12_2	0.950000
C13_2-C14_2	1.411(4)	C13_2-H13_2	0.950000
C14_2-C15_2	1.370(4)	C14_2-H14_2	0.950000
C15_2-C15A_2	1.398(3)	C15_2-H15_2	0.950000
C16_2-H16A_2	0.990000	C16_2-H16B_2	0.990000

Table S3. Crystallographic Table of Bond Angles (°) for **L^1**

N2_1-N1_1-C7A_1	111.3(2)	N2_1-N1_1-C8_1	120.11(17)
C7A_1-N1_1-C8_1	128.59(18)	C3_1-N2_1-N1_1	106.0(2)
N2_1-C3_1-C3A_1	111.9(2)	N2_1-C3_1-H3_1	124.100000
C3A_1-C3_1-H3_1	124.100000	C7A_1-C3A_1-C4_1	119.3(2)
C7A_1-C3A_1-C3_1	104.3(2)	C4_1-C3A_1-C3_1	136.4(3)
C5_1-C4_1-C3A_1	118.0(3)	C5_1-C4_1-H4_1	121.000000
C3A_1-C4_1-H4_1	121.000000	C4_1-C5_1-C6_1	121.7(2)
C4_1-C5_1-H5_1	119.200000	C6_1-C5_1-H5_1	119.200000
C7_1-C6_1-C5_1	122.2(3)	C7_1-C6_1-H6_1	118.900000
C5_1-C6_1-H6_1	118.900000	C6_1-C7_1-C7A_1	116.0(3)
C6_1-C7_1-H7_1	122.000000	C7A_1-C7_1-H7_1	122.000000
N1_1-C7A_1-C3A_1	106.6(2)	N1_1-C7A_1-C7_1	130.5(2)
C3A_1-C7A_1-C7_1	122.9(2)	N1_1-C8_1-N1_1#2	113.5(3)
N1_1-C8_1-H8A_1	108.900000	N1_1#2-C8_1-H8A_1	108.900000
N1_1-C8_1-H8B_1	108.900000	N1_1#2-C8_1-H8B_1	108.900000

H8A_1-C8_1-H8B_1	107.700000	C15A_2-N9_2-N10_2	111.8(2)
C15A_2-N9_2-C16_2	128.91(18)	N10_2-N9_2-C16_2	119.29(18)
C11_2-N10_2-N9_2	105.4(2)	N10_2-C11_2-C11A_2	112.5(2)
N10_2-C11_2-H11_2	123.800000	C11A_2-C11_2-H11_2	123.800000
C12_2-C11A_2-C15A_2	119.1(2)	C12_2-C11A_2-C11_2	136.9(3)
C15A_2-C11A_2-C11_2	104.0(2)	C13_2-C12_2-C11A_2	118.8(3)
C13_2-C12_2-H12_2	120.600000	C11A_2-C12_2-H12_2	120.600000
C12_2-C13_2-C14_2	121.1(3)	C12_2-C13_2-H13_2	119.500000
C14_2-C13_2-H13_2	119.500000	C15_2-C14_2-C13_2	122.0(3)
C15_2-C14_2-H14_2	119.000000	C13_2-C14_2-H14_2	119.000000
C14_2-C15_2-C15A_2	116.8(3)	C14_2-C15_2-H15_2	121.600000
C15A_2-C15_2-H15_2	121.600000	N9_2-C15A_2-C15_2	131.6(2)
N9_2-C15A_2-C11A_2	106.3(2)	C15_2-C15A_2-C11A_2	122.2(2)
N9_2-C16_2-N9_2#1	113.4(3)	N9_2-C16_2-H16A_2	108.900000
N9_2#1-C16_2-H16A_2	108.900000	N9_2-C16_2-H16B_2	108.900000
N9_2#1-C16_2-H16B_2	108.900000	H16A_2-C16_2-H16B_2	107.700000

Symmetry transformations used to generate equivalent atoms:
#1 -x+1, y, -z
#2 -x+1, y, -z+1

Table S4. Testing solvents for conversion of 1*H*-indazole to bis(indazol-1-yl)methane using 10% $CoCl_2 \cdot 6H_2O$ and 24 hours near the boiling point of each solvent as listed.

Solvent	Temperature	Dimer Formation?
Dimethylsulfoxide	175 °C	Yes
Dimethylformamide	153 °C	No
Toluene + DMSO	120 °C	No
Toluene	115 °C	No
Dibromomethane	100 °C	No
Acetonitrile	82 °C	No
Ethanol	78 °C	No
Tetrahydrofuran	66 °C	No
Dichloromethane	40 °C	No
37% Formaldehyde in water	40 °C[a]	No[b]

[a] In the case of the formaldehyde solution, a slight amount of heat was necessary to solubilize the starting material indazole. However, care was taken to keep this reaction well below the flashpoint of the solution and therefore well below the boiling point.

[b] In all other solvents, only the 1*H*-indazole starting material was detected. In the case of the formaldehyde solution, a complex mixture of products was obtained but no dimer or 1*H*-indazole was detected by ^1H NMR.

References:

(1) Elguero, J.; Fruchier, A.; Tjiou, E. M.; Trofimenko, S. 13C NMR of Indazoles. *Chemistry of Heterocyclic Compounds* **1995**, *31*, 1006–1026.

(2) Lukin, K.; Hsu, M. C.; Fernando, D.; Leanna, M. R. New Practical Synthesis of Indazoles via Condensation of O-Fluorobenzaldehydes and Their O-Methyloximes with Hydrazine. *Journal of Organic Chemistry* **2006**, *71* (21), 8166–8172. https://doi.org/10.1021/jo0613784.

(3) Juliá, S.; Sala, P.; del Mazo, J.; Sancho, M.; Ochoa, C.; Elguero, J.; Fayet, J. -P; Vertut, M. -C. N-polyazolylmethanes. 1. Synthesis and Nmr Study of N,N′-diazolylmethanes. *Journal of Heterocyclic Chemistry*. 1982, pp 1141–1145. https://doi.org/10.1002/jhet.5570190531.

(4) Pettinari, C.; Marinelli, A.; Marchetti, F.; Ngoune, J.; Galindo, A.; Álvarez, E.; Gómez, M. Synthesis and Coordination Chemistry of Two N2-Donor Chelating Di(Indazolyl)Methane Ligands: Structural Characterization and Comparison of Their Metal Chelation Aptitudes. *Inorganic Chemistry* **2010**, *49* (22), 10543–10556. https://doi.org/10.1021/ic101577k.
